# Synthetic Marijuana Induced Acute Nonischemic Left Ventricular Dysfunction

**DOI:** 10.1155/2016/9625758

**Published:** 2016-03-28

**Authors:** Moustafa Elsheshtawy, Priatharsini Sriganesh, Vasudev Virparia, Falgun Patel, Ashok Khanna

**Affiliations:** Division of Cardiology, Department of Medicine, Coney Island Hospital, Brooklyn, NY 11235, USA

## Abstract

Synthetic marijuana is an uptrending designer drug currently widely spread in the US. We report a case of acute deterioration of nonischemic left ventricular dysfunction after exposure to synthetic marijuana. This case illustrates the importance of history taking in cardiac patients and identifies a negative cardiovascular effect of synthetic marijuana known as K2, not yet well detected by urine toxicology screening tools.

## 1. Introduction

Synthetic cannabinoids (marijuana), also known as K2, were first reported in the US in 2008. Synthetic cannabinoids exert their potent effect on the myocardium either via interacting with different receptors or through excess catecholamine release. Deployed impact can lead to major cardiovascular events including myocardial infarction and fatal arrhythmias. As of today, routine toxicology screening tests used to detect synthetic marijuana are not available in most hospitals. Development of an appropriate drug screen to detect synthetic cannabinoids is complicated by the compound variability in drug combinations. This suggests that healthcare providers who exclude drug-induced toxicities based on a negative drug screen result will continue to underestimate the impact of synthetic marijuana on health.

## 2. Case Report

A 50-year-old man came to the emergency department complaining of sudden onset shortness of breath and choking sensation. Symptoms started a few minutes after smoking a synthetic marijuana joint. The patient also stated that he has been coughing with yellow mucus sometimes tinged with blood streaks for five days prior to presentation. However, he denied any chest pain, diaphoresis, or dizziness.

Patient's past medical history was significant for mild systolic dysfunction, hypertension, and residual right sided hemiparesis and rigidity following motor vehicle accident many years ago. He denies any history of heart attacks in the past and was never diagnosed with atherosclerotic coronary vascular disease. The patient stated that he has been smoking one pack of cigarettes a day for 35 years and admitted to smoking natural marijuana for recreation occasionally. The patient stated that, on the day of presentation, he tried to smoke a synthetic marijuana joint for the first time. The patient was prescribed angiotensin converting enzyme inhibitor by his primary doctor, but it was unclear whether he was on any beta-blockers.

Vital signs on admission were stable except for an elevated heart rate ranging between 100 and 110 beats per minute. Physical exams demonstrated bilateral rales mainly basal and more on the right side. Mild expiratory rhonchi were also noted on both lung fields. Laboratory findings were significant for elevated pronatriuretic peptide (5207 pg/ml) and mild leukocytosis (17.7 K/*μ*l). Other laboratory tests including thyroid function tests, three sets of cardiac enzymes, and screening urine toxicology were all normal.

**Figure 1:**
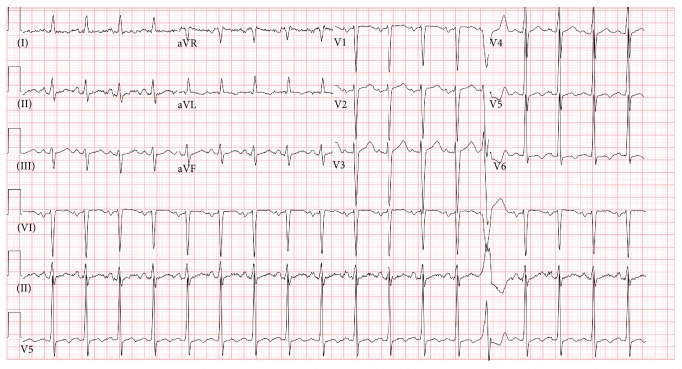
ECG on admission showing sinus tachycardia with a premature ventricular contraction.

ECG on admission showed sinus tachycardia with premature ventricular contractions (PVCs), left atrial enlargement, and left ventricular hypertrophy with prolonged QTc (488 msec). ST and T wave nonspecific changes were also noted on the lateral leads of ECG (Figure 1).

**Figure 2:**
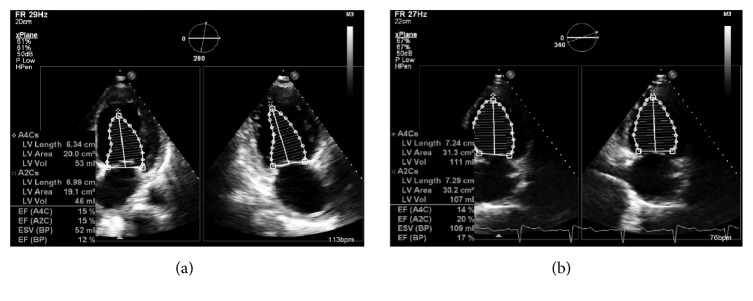
Echocardiography demonstrating decreased ejection fraction on admission (a) and two months later (b).

**Figure 3:**
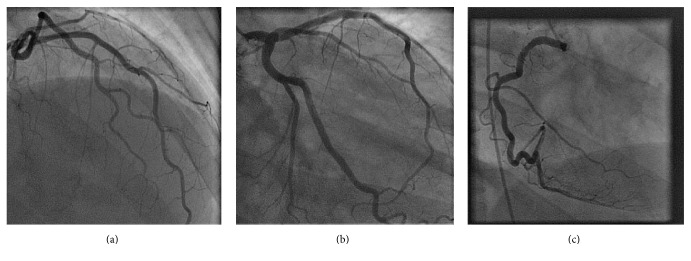
Coronary catheter angiogram revealing normal coronary vessels. Left circumflex artery (a), left main artery (b), and right main coronary artery (c) are patent.

Chest X-ray revealed pulmonary venous congestion associated with bilateral pleural effusions. Computed tomography (CT) of the chest with contrast was diagnostic of cardiogenic pulmonary edema and the patient was admitted for management of congestive heart failure exacerbation. The patient was started on diuretics and antibiotics for possible aspiration pneumonia. An echocardiogram showed severely reduced left ventricular function with an ejection fraction (EF) of 10–15% associated with global hypokinesis (Figure 2(a)) compared to an EF of 32% reported by the patient's private cardiologist a month before admission. The patient showed improvement clinically and radiologically after two days of treatment and was restarted on angiotensin converting enzyme inhibitor (ACEI) and beta-blocker medications. Coronary catheter angiogram did not reveal any coronary vessel blockade (Figure 3) and the patient received life vest upon discharge. A follow-up echocardiogram recorded 2 months later revealed stable reduced left ventricular function with an ejection fraction (EF) of 10–15% as previously noted (Figure 2(b)).

## 3. Discussion

Synthetic marijuana is a chemically unrelated analog of the natural cannabinoid, delta-9-tetrahydrocannabinol (THC), the active principle of cannabis [1]. Up to 7000 cases of synthetic marijuana intoxication are reported in the US annually [2]. Members of the “Spice” family are up to 800 times more potent agonists than THC. They act mainly on the two types of cannabinoid receptors (CB), with type 1 mainly present in the CNS causing the psychoactive state. Most synthetic cannabinoids, whether smoked or infused orally, do not show cross-reactivity to THC or its metabolites and are not detected on antibody-based drug test assays like rapid urine drug screens [1].

Tachycardia is the most common symptom reported from synthetic marijuana intoxication [2]. Animal and in vitro studies varied in establishing cannabis' effect on the myocardium either through brain centers or via exerting a direct effect on the vascular tone.

Marijuana was shown to augment left ventricular function and increase heart rate through excessive catecholamine release for few hours after exposure [3]. THC was shown to increase cardiac output by up to 30% and heart rate by 20–100% in a dose-dependent way with consequent reduction in left ventricular ejection time [3, 4]. THC can also facilitate atrioventricular node conduction and decrease peripheral vascular resistance [3]. These observed effects increase the oxygen demand of the myocardium and can trigger myocardial infarction and have been linked to ventricular tachycardia and ventricular fibrillation in some cases [4, 5]. In a large epidemiologic study, marijuana was found to increase myocardial infarction 4.8-fold in the first 1 hour following marijuana use compared to periods of nonuse (95% confidence interval: 2.4–9.5, *P* < 0.001) [5].

In one study, endocannabinoid anandamide (AEA) inhibited the function of voltage-dependent sodium (Na^+^) and L-type calcium (Ca^2+^) channels in rat ventricular myocytes independent of CB_1_ and CB_2_ receptor activation [6]. CB_1_ receptor activation resulted in a negative chronotropic effect and decreased force of isolated heart contractions. Observed effect was less pronounced in vitro than in vivo and was independent of the autonomic nervous system [7].

In our case, we report a sudden acute deterioration of left ventricular function following exposure to synthetic marijuana. Establishing this connection is based on history given by the patient and after ruling out ischemic heart disease as a possible cause. Our patient did not show any signs of myocardial ischemic changes and coronary angiogram was normal. Since it is expected that the ejection fraction would have partially recovered after two months if the synthetic cannabinoid was the only reason to blame, it is also unclear if this patient ever stopped abusing the substance. Linking K2 abuse solely to the myocardium depression in our case has been a challenge especially in the absence of urine toxicology test quantifying the substance level. However, establishing the relationship between K2 and its negative sequelae on the myocardium will require further studies to conclude such effects. To our knowledge, this is the first reported case in adults of acute nonischemic left ventricular dysfunction after exposure to synthetic marijuana. This case identifies a negative cardiovascular effect of synthetic marijuana known as “Spice.”
